# Self-reported data for mental workload modelling in human-computer interaction and third-level education

**DOI:** 10.1016/j.dib.2020.105433

**Published:** 2020-03-19

**Authors:** Lucas Rizzo, Luca Longo

**Affiliations:** a Technological University Dublin, Republic of Ireland; b Technological University Dublin, Republic of Ireland

**Keywords:** Knowledge-based systems, Fuzzy reasoning, Expert systems, Mental workload, Automated reasoning, Argumentation theory

## Abstract

Mental workload (MWL) is an imprecise construct, with distinct definitions and no predominant measurement technique. It can be intuitively seen as the amount of mental activity devoted to a certain task over time. Several approaches have been proposed in the literature for the modelling and assessment of MWL. In this paper, data related to two sets of tasks performed by participants under different conditions is reported. This data was gathered from different sets of questionnaires answered by these participants. These questionnaires were aimed at assessing the features believed by domain experts to influence overall mental workload. In total, 872 records are reported, each representing the answers given by a user after performing a task. On the one hand, collected data might support machine learning researchers interested in using predictive analytics for the assessment of mental workload. On the other hand, data, if exploited by a set of rules/arguments (as in [3]), may serve as knowledge-bases for researchers in the field of knowledge-based systems and automated reasoning. Lastly, data might serve as a source of information for mental workload designers interested in investigating the features reported here for mental workload modelling. This article was co-submitted from a research journal “An empirical evaluation of the inferential capacity of defeasible argumentation, non-monotonic fuzzy reasoning and expert systems” [3]. The reader is referred to it for the interpretation of the data.

Specifications table

**Table:** 

Subject	Artificial Intelligence
Specific subject area	Knowledge-based systems. Human mental workload.
Type of data	Table
How data were acquired	Survey.
Data format	Raw
Parameters for data collection	Data was collected according to a set of tasks believed to impose different levels of mental workload on participants.
Description of data collection	One part of data was collected through a set of surveys applied to students who attended classes at the Technological University Dublin (Table 1). The other part of data was also collected through a set of surveys, but this time answered by volunteers who performed a set of designed web-based tasks (Table 7).
Data source location	City/Town/Region: Dublin Country: Ireland
Data accessibility	With the article and as a supplementary attachment.
Related research article	Author's name: Lucas Rizzo and Luca Longo Title: An empirical evaluation of the inferential capacity of defeasible argumentation, non-monotonic fuzzy reasoning and expert systems [3] Journal: Expert Systems with Applications.

## Value of the data

• These datasets provide the answers related to a set of questionnaires proposed in the literature of MWL (Tables 2, 3 and 5) aimed at assessing the mental workload imposed on participants by a set of designed tasks (Tables 1 and 7). In total 872 records are reported, each representing the answers given by a user after performing a task.
• These are important to the field of human mental workload and knowledge representation and reasoning. They may instantiate knowledge-bases created by researches in the field of knowledge-based systems and automated reasoning. It might also support machine learning researchers interested in using predictive analytics for the assessment of mental workload. Lastly, they may serve as a source of information for mental workload designers interested in investigating the features reported here for mental workload modelling.
• They may serve as baselines for comparison against newly developed models of similar purpose. Reported values by three state-of-the-art measurement techniques of mental workload are also displayed: the NASA – Task Load Index [1], the Workload Profile [4] and the Raw Task Load Index [5]. A self-report indicating the participants perceived mental workload is also provided. This can be applied for triangulation purposes when developing new methods of mental workload assessment.

## 1 Data

In this section three datasets in the field of human mental workload are described. These datasets are built upon subjective measures of mental workload. In other words, they rely on the subjective feedback - in this case questionnaires - provided by humans engaging with an underlying task. These questionnaires (Tables 2, 3 and 5) are proposed in the literature of MWL [1,2,4]. For each dataset these are defined:

1. A set of tasks performed (Tables 1 or 7).
2. A set of questionnaires proposed in the literature of MWL aimed at measuring the mental workload imposed by tasks in 1.
3. A dataset in *.csv* format containing one row per answers of questions in 2 given by each participant who performed one task listed in 1.

In [3], these datasets are also employed for the construction of fuzzy rule-based systems and argument-based systems. Such systems are built upon a set of rules/arguments able to infer a numerical MWL scalar from the data described in this article.

### 1.1 Dataset A

The set of performed tasks in this dataset is listed in Table 1. The set of questionnaires employed for measuring the MWL imposed by these tasks is listed in Table 2 and 3. Finally, the description of collected associated to this dataset is listed in Table 4.

**Table 1:** List of tasks given in the form of third-level classes to students at Technological University Dublin, School of Computer Science, Dublin, Ireland. Four topics of the module ‘Research Methods’ in the Master of Science were delivered by three distinct approaches.

Topics	1. Science
	2. Scientific method
	3. Research planning
	4. Literature review

Instructional condition	1. Traditional direct instruction, using slides projected to a white board;
	2. Multimedia video of content. Transformation of the content of the slides of 1 into a multimedia video projected to a white board;
	3. Constructivist collaborative activity added to 2.

**Table 2:** The questionnaire of the Nasa Task Load Index [1].

Feature	Question
Mental demand	How much mental and perceptual activity was required (e.g. thinking, deciding, calculating, remembering, looking, searching, etc.)? Was the task easy or demanding, simple or complex, exacting or forgiving?
Physical demand	How much physical activity was required (e.g. pushing, pulling, turning, controlling, activating, etc.)? Was the task easy or demanding, slow or brisk, slack or strenuous, restful or laborious?
Temporal demand	How much time pressure did you feel due to the rate or pace at which the tasks or task elements occurred? Was the pace slow and leisurely or rapid and frantic?
Effort	How hard did you have to work (mentally and physically) to accomplish your level of performance?
Performance	How successful do you think you were in accomplishing the goals, of the task set by the experimenter (or yourself)? How satisfied were you with your performance in accomplishing these goals?
Frustration	How insecure, discouraged, irritated, stressed and annoyed versus secure, gratified, content, relaxed and complacent did you feel during the task?

**Table 3:** The questionnaire of the pairwise comparison procedure of the Nasa Task Load Index [1].

Pair	Factor 1		Factor 2
1	Temporal demand	⬜ OR ⬜	Frustration
2	Performance	⬜ OR ⬜	Mental
3	Mental demand	⬜ OR ⬜	Physical demand
4	Frustration	⬜ OR ⬜	Performance
5	Temporal demand	⬜ OR ⬜	Effort
6	Physical demand	⬜ OR ⬜	Frustration
7	Performance	⬜ OR ⬜	Temporal demand
8	Mental demand	⬜ OR ⬜	Effort
9	Physical demand	⬜ OR ⬜	Temporal demand
10	Frustration	⬜ OR ⬜	Effort
11	Physical demand	⬜ OR ⬜	Performance
12	Temporal demand	⬜ OR ⬜	Mental demand
13	Effort	⬜ OR ⬜	Physical demand
14	Frustration	⬜ OR ⬜	Performance
15	Performance	⬜ OR ⬜	Mental demand

**Table 4:** Description of columns in attached data table in supplementary attachment A.

Column header	Description
class_description	Type of class attended by students of the “Research methods” module (Table 3). Four possible values: literature_review, planning_research, science and the_scientific_method
delivery_method	Delivery method employed during the class (Table 3). Three possible values: pdf, video and video_and_collaborative_activity
nationality	Nationality of the participant
Age	Age in years of the participant
Date	Date relative to the day the record was collect
mental_workload	Self-assessment mental workload reported by Fig. 1. Integer between 1 and 20
NASA_TLX	The NASA-TLX score [1]. Real number between 0 and 100.
mental_demand	Mental demand reported according to question of Table 2. Integer between 1 and 20.
physical_demand	Physical demand reported according to question of Table 2. Integer between 1 and 20.
temporal_demand	Temporal demand reported according to question of Table 2. Integer between 1 and 20.
performance	Performance reported according to question of Table 2. Integer between 1 and 20.
frustration	Frustration reported according to question of Table 2. Integer between 1 and 20.
effort	Effort reported according to question of Table 2. Integer between 1 and 20.
factor1_vs_factor2 (columns N to AB)	Pairwise comparison of the 15 pairs of Table 3. Possible values are 0 (factor 1 was chosen) or 1 (factor 2 was chosen).

### 1.2 Dataset B

Tasks employed for the construction of this dataset are the same ones described in Table 1. Remaining information for the construction of this dataset is listed below. The questionnaire employed for measuring the MWL imposed by these tasks is listed in Table 5, while the description of collected data is listed in Table 6

**Table 5:** Features and experimental study questionnaire designed by Longo [2].

Feature [Source]	Question
Mental demand [1]	How much mental and perceptual activity was required (e.g., thinking, deciding, calculating, remembering, looking, searching, etc.)? Was the task easy (low mental demand) or complex (high mental demand)?
Temporal demand [1]	How much time pressure did you feel due to the rate or pace at which the tasks or task elements occurred? Was the pace slow and leisurely (low temporal demand) or rapid and frantic (high temporal demand)?
Effort [1]	How much conscious mental effort or concentration was required? Was the task almost automatic (low effort) or it required total attention (high effort)?
Performance [1]	How successful do you think you were in accomplishing the goal of the task? How satisfied were you with your performance in accomplishing the goal?
Frustration [1]	How secure, gratified, content, relaxed and complacent (low psychological stress) versus insecure, discouraged, irritated, stressed and annoyed (high psychological stress) did you feel during the task?
Physical demand [1]	How much physical activity was required (e.g. pushing, pulling, turning, controlling, activating, etc.)? Was the task easy or demanding, slow or brisk, slack or strenuous, restful or laborious?
Solving and deciding [4]	How much attention was required for activities like remembering, problem-solving, decision-making and perceiving (e.g. detecting, recognizing and identifying objects)?
Selection of response [4]	How much attention was required for selecting the proper response channel and its execution? (manual - keyboard/mouse, or speech - voice)
Task and space [4]	How much attention was required for spatial processing (spatially pay attention around you)?
Verbal material [4]	How much attention was required for verbal material (eg. reading or processing linguistic material or listening to verbal conversations)?
Visual resources [4]	How much attention was required for executing the task based on the information visually received (through eyes)?
Auditory resources [4]	How much attention was required for executing the task based on the information auditorily received (ears)?
Manual Response [4]	How much attention was required for manually respond to the task (e.g. keyboard/mouse usage)?
Speech response [4]	How much attention was required for producing the speech response (e.g. engaging in a conversation or talk or answering questions)?
Context bias [2]	How often interruptions on the task occurred? Were distractions (mobile, questions, noise, etc.) not important (low context bias) or did they influence your task (high context bias)?
Past knowledge [2]	How much experience do you have in performing the task or similar tasks on the same website?
Skill [2]	Did your skills have no influence (low) or did they help to execute the task (high)?
Motivation [2]	Were you motivated to complete the task?
Parallelism [2]	Did you perform just this task (low parallelism) or were you doing other parallel tasks (high parallelism) (e.g. multiple tabs / windows / programs)?
Arousal [2]	Were you aroused during the task? Were you sleepy, tired (low arousal) or fully awake and activated (high arousal)?
Task difficult [2]	1 / 8 * [(solving and deciding) + (auditory resources) + (manual response) + (speech response) + (selection of response) + (task and space) + (verbal material) + (visual resources)]

**Table 6:** Description of columns in attached data table in supplementary attachment B.

Column header	Description
class_description	Four possible values (Table 3): literature_review, planning_research, science and the_scientific_method
delivery_method	Three possible values (Table 3): pdf, video and video_and_collaborative_activity
Date	Date relative to the day the record was collect
nationality	Nationality of the participant
Age	Age in years of the participant
mental_workload	Self-assessment mental workload reported by Fig. 1. Integer between 1 and 20
MentalDemand	Mental demand reported according to question of Table 5. Integer between 1 and 20.
Parallelism	Parallelism reported according to question of Table 5. Integer between 1 and 20.
TemporalDemand	Temporal demand reported according to question of Table 5. Integer between 1 and 20.
ManualResponse	Manual response reported according to question of Table 5. Integer between 1 and 20.
VisualResources	Visual resources reported according to question of Table 5. Integer between 1 and 20.
Effort	Effort reported according to question of Table 5. Integer between 1 and 20.
SolvingAndDeciding	Solving and deciding reported according to question of Table 5. Integer between 1 and 20.
Frustration	Frustration reported according to question of Table 5. Integer between 1 and 20.
ContextBias	Context bias reported according to question of Table 5. Integer between 1 and 20.
TaskAndSpace	Task and space reported according to question of Table 5. Integer between 1 and 20.
Motivation	Motivation reported according to question of Table 5. Integer between 1 and 20.
VerbalMaterial	Verbal material reported according to question of Table 5. Integer between 1 and 20.
Skill	Skill reported according to question of Table 5. Integer between 1 and 20.
AuditoryResources	Auditory resources reported according to question of Table 5. Integer between 1 and 20.
PhysicalDemand	Physical demand reported according to question of Table 5. Integer between 1 and 20.
Selection Of Response	Selection of response reported according to question of Table 5. Integer between 1 and 20.
SpeechResponse	Speech response reported according to question of Table 5. Integer between 1 and 20.
PastKnowledge	Past knowledge reported according to question of Table 5. Integer between 1 and 20.
Arousal	Arousal reported according to question of Table 5. Integer between 1 and 20.
Performance	Performance reported according to question of Table 5. Integer between 1 and 20.
TaskDifficult	Task difficult reported according to question of Table 5. Real number between 1 and 20.
RAW_TLX	The Raw TLX score [5]. Real number between 0 and 100.
WorkloadProfile	The Workload Profile score [4]. Real number between 0 and 100.

### 1.3 Dataset C

The set of tasks performed by participants in the construction of this dataset is listed in Table 7. Questionnaires answered by participants after performing a task can be seen in Tables 5 and 3. Remaining information for the construction of this dataset is listed below. The description of data collected is listed in Table 8.

**Table 7:** List of seeking web-based tasks of varying difficulty and demand. These were first designed in [2].

Task	Description	Task condition	Web-site
1	Find out how many people live in Sidney	Simple search	Wikipedia
2	Read simple.wikipedia.org/wiki/Grammar	No goals, no time pressure	Wikipedia
3	Find out the difference (in years) between the year of the foundation of the Apple Computer Inc. and the year of the 14th FIFA world cup	Dual-task and mental arithmetical calculations	Google
4	Find out the difference (in years) between the foundation of the Microsoft Corp. and the year of the 23rd Olympic games	Dual-task and mental arithmetical calculations	Google
5	Find out the year of birth of the 1st wife of the founder of playboy	Single task + time pressure (2-min limit). Each 30 secs user is warned of time left	Google
6	Find out the name of the man (interpreted by Johnny Deep) in the video www.youtube.com/watch?v=FfTPS-TFQ_c	Constant demand on visual and auditory modalities. Participant can replay the video if required	YouTube
7	a) Play the song www.youtube.com/watch?v=Rb5G1eRIj6c While listening to it, b) ind out the result of the polynomial equation p(x), with *x* = 7 contained in the wikipedia article http://it.wikipedia.org/wiki/Polinomi	Demand on visual modality and inference on auditory modality. The song is extremely irritating	Wikipedia
8	Find out how many times Stewie jumps in the video www.youtube.com/watch?v=TSe9gbdkQ8s	Demand on visual resource + external interference: user is distracted twice and can replay video	YouTube
9	Find out the age of the blue fish in the video www.youtube.com/watch?v=H4BNbHBcnDI	Demand on visual and auditory modality, plus time-pressure: 150 s limit. User can replay the video. There is no answer.	YouTube

**Table 8:** Description of columns in attached data table in supplementary attachment C.

Column header	Description
TaskNumber	Number of the task performed by volunteer. List of tasks can be seen in Table 4.
InterfaceVersion	Interface of the website in which the task was performed. Two options are possible: original and modified.
CompletionTimeInSeconds	Time taken for completion of the task.
GenderCat	Gender of the volunteer: male or female.
BornYear	The year of birth of the volunteer
LangCat	The main language spoken by the user (however all the users were almost fluent in English). Options are: Chinese, Czech, English, French, German, Italian, Polish, Portuguese, Spanish and other.
ContextBias	Context bias reported according to question of Table 5. Integer between 1 and 100
PastKnowledge	Past knowledge reported according to question of Table 5. Integer between 1 and 100
Skill	Skill reported according to question of Table 5. Integer between 1 and 100
Motivation	Motivation reported according to question of Table 5. Integer between 1 and 100
Parallelism	Parallelism reported according to question of Table 5. Integer between 1 and 100
Arousal	Arousal reported according to question of Table 5. Integer between 1 and 100
SolvingAndDeciding	Context bias reported according to question of Table 5. Integer between 1 and 100
SelectionOfResponse	Solving and deciding reported according to question of Table 5. Integer between 1 and 100
VerbalMaterial	Verbal material reported according to question of Table 5. Integer between 1 and 100
VisualResources	Visual resources reported according to question of Table 5. Integer between 1 and 100
AuditoryResources	Auditory resources reported according to question of Table 5. Integer between 1 and 100
ManualResponse	Manual response according to question of Table 5. Integer between 1 and 100
SpeechResponse	Speech response reported according to question of Table 5. Integer between 1 and 100
TaskDifficult	Task difficult reported according to question of Table 5. Integer between 1 and 100
MentalDemand	Mental demand reported according to question of Table 5. Integer between 1 and 100
TemporalDemand	Temporal demand reported according to question of Table 5. Integer between 1 and 100
Frustration	Frustration reported according to question of Table 5. Integer between 1 and 100
Effort	Effort reported according to question of Table 5. Integer between 1 and 100
Performance	Performance reported according to question of Table 5. Integer between 1 and 100
PhysicalDemand	Physical demand was considered 0 for all instances of this dataset.
factor1_vs_factor2 (columns AB to AP)	Pairwise comparison of the 15 pairs of Table 3. Possible values are 0 (factor 1 was chosen) or 1 (factor 2 was chosen).
NasaTLXRaw	The Raw TLX score [5]. Real number between 0 and 100.
NASATLX	The Workload Profile score [4]. Real number between 0 and 100.
WP	The NASA-TLX score [1]. Real number between 0 and 100.

## 2 Experimental design, materials, and methods

**Fig. 1:**
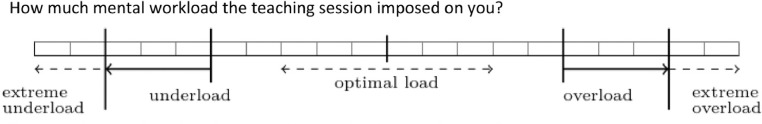
Baseline self-reporting measure of Mental Workload [3].

Mental workload (MWL) is an imprecise construct, with distinct definitions and no predominant measurement technique. It can be intuitively seen as the amount of mental activity devoted to a certain task over time. Several approaches have been proposed in the literature [1,2,4,5] for the modelling and assessment of MWL. Data reported here is relative to two sets of tasks (third-level classes in Table 1 and seeking web-based information in Table 7) performed by several participants. Subjects were briefed about the study and they were requested to sign a consent form that included data protection and treatment. Privacy and anonymity of participants were in all respects protected by the authors. The goal was to collect the information asked in Fig. 1 and in Tables 2, 3 and 5. These contained features believed by domain experts to influence overall imposed mental workload by performed tasks. MWL is an undefined psychological construct. Therefore, the goal of the data is to help scholars to understand and develop new measurements techniques of MWL. No participant performed/answered the same task/questionnaire more than once, avoiding ambiguous data. Hence, each record can be employed for a case-by-case analysis of the MWL imposed by the respective task.

### 2.1 Third-level classes at Dublin Institute of Technology

Students attended third level classes in the Technological University Dublin and filled either questionnaires in Tables 2 and 3 or only the questionnaire in Table 7 after each class (Table 1). The set of questionnaires were related to the features perceived by different mental workload designers to influence the imposed MWL by the performed task. In Tables 2 and 3 only features of the NASA-TLX [1] measurement technique were being investigated, while in Table 7 a larger set of features [2] was being considered for MWL modelling and assessment. Therefore, two distinct sets of data were generated and reported in the data tables of supplementary attachments A and B. In total, students were from 24 distinct countries (age 19–74, mean 30.9, std = 7.63). In general, four topics of the module ‘Research Methods’ were delivered in three different forms (Table 1) during the semesters of the academic terms 2015–2018. Some group of students received the first instructional condition (PDF slides presented by lecturer to students), some received the second instructional condition (same content of PDF slides presented through video and no lecturer), and some received the third instructional condition (same as the second instructional condition with a collaborative group activity added at the end). The number of students who attended each class is described in Table 9.

**Table 9:** Number of students across topics and delivery methods.

Topic	Duration (mins)	Delivery method		
		1	2	3
Science	[18, 62]	31	70	19
Scientific method	[20, 46]	39	36	41
Research planning	[10, 68]	43	45	41
Literature review	[18, 57]	41	43	18

### 2.2 Information seeking web-based tasks

Nine information seeking web-based tasks of varying difficulty and demand (Table 7), were performed by participants over three websites: Google, Wikipedia and YouTube. These websites were selected due to their popularity and assumption that participants were familiar with their interfaces. The original interface of each web-site was slightly manipulated to impose different MWL demands on participants interacting with them, leading to 9 tasks on the original websites and 9 tasks on the modified websites (18 in total). 46 volunteers performed all the tasks in a random order on different days, over 2 or 3 sessions of approximately 45/70 min each. Afterwards, the questions of Tables 3 and 7 were answered using a paper-based scale in the range [0..100] ∈ א, partitioned in 3 regions delimited at 33 and 66. 405 valid instances were generated.

## Appendix Supplementary materials

Image, application 1
